# The N. Y. Observer

**Published:** 1874-12

**Authors:** 


THE NEW YORK OBSERVER.—The New York Observer still continues to be as interesting and valuable as ever, under the editorial management of Dr. PRIME, it has been for years considered as one of the best religious papers published. It is published weekly on the following terms: Yearly in advance $3.15. To two NEW subscribers $5.30.

